# The Long and Short of It: The Role of Telomeres in Fetal Origins of Adult Disease

**DOI:** 10.1155/2012/638476

**Published:** 2012-10-03

**Authors:** Stephanie E. Hallows, Timothy R. H. Regnault, Dean H. Betts

**Affiliations:** ^1^Department of Physiology and Pharmacology, University of Western Ontario, Ontario, London, ON, Canada N6A 5C1; ^2^Department of Obstetrics and Gynaecology, University of Western Ontario, Ontario, London, ON, Canada N6H 5W9; ^3^Children's Health Research Institute, Lawson Health Research Institute, London, ON, Canada N6C 2V5

## Abstract

Placental insufficiency, maternal malnutrition, and other causes of intrauterine growth restriction (IUGR) can significantly affect short-term growth and long-term health. Following IUGR, there is an increased risk for cardiovascular disease and Type 2 Diabetes. The etiology of these diseases is beginning to be elucidated, and premature aging or cellular senescence through increased oxidative stress and DNA damage to telomeric ends may be initiators of these disease processes. This paper will explore the areas where telomere and telomerase biology can have significant effects on various tissues in the body in IUGR outcomes.

## 1. Intrauterine Growth Restriction and Placental Insufficiency

 The World Health Organization (WHO) estimates that the incidence of infants born low birth weight (LBW) in North America is approximately 7% [1] and is commonly characterized as a birth weight under 2500 g. A further sub classification within the LBW classification is that of intrauterine growth restriction (IUGR) where a fetus fails to reach its genetic growth potential, as a result of a compromised intrauterine environment and is generally defined as being less than the 3rd percentile of normal birth weight. IUGR can be caused by a heterogeneous mix of fetal, placental and maternal factors. Fetal factors include genetic abnormalities, multiple gestation, and infections [2], while maternal contributing factors for IUGR include malnutrition, drug intake, hypertension, Type I or gestational diabetes, and persistent hypoxia due to cardiovascular disease or high altitude [2]. Placental insufficiency is a common cause of IUGR, accounting for ~60% of IUGR and includes reduced placental development, abnormal trophoblast invasion into the maternal decidua, placenta previa, and placental infarcts [3, 4]. Human studies and animal models of placental insufficiency have demonstrated a decreased rate of nutrient transfer across the placenta. Specifically, IUGR fetuses are characterized by alterations in oxygen supply [5–7], glucose and amino acid supply [8–10], and with increased fetal triglycerides [11, 12]. Due to the lack of oxygen and altered nutrient balance, the fetus redirects these scarce resources to the brain, heart, and adrenal glands, leaving other tissues in the body more severely growth restricted, resulting in asymmetric IUGR [13, 14]. This redistribution of nutritional supplies leads to a decrease in muscularity and an increase in the percentage of body fat in these infants that persists throughout childhood and adult life [15, 16] and is commonly associated with changes in insulin sensitivity and other markers of the metabolic syndrome [17, 18]. These observations and others set the stage for the idea that changes in growth during *in utero* life may predispose offspring to increased risk of disease in later life, or the concept of the developmental origins of health and disease (DOHaD).

## 2. Developmental Origins of Health and Disease

 LBW infants primarily present an increased risk for perinatal morbidity and mortality [19]. However, through the work of David Barker and colleagues, the concept that there further exists a relationship between birth weight and an increased risk for developing diseases including coronary heart disease, Type 2 Diabetes, and hypertension in later life has been generally accepted as a secondary concern for LBW infants [20, 21]. Since the early observations, this relationship between low birth weights, followed by a rapid catch up growth leading to increased risk of adult disease has been reported in a number of human population studies and in many animal models of IUGR [22, 23]. 

Barker and colleagues theorized that there are critical periods during development when the fetus adapts and is programmed to its *in utero* surroundings, and after which the fetuses phenotype is established [24]. This is the basis of the “thrifty phenotype” hypothesis, where there is a mismatch between the intrauterine environment the fetuses encounters, and the exuterine environment an individual grows up in [25]. This can cause a relative over compensation in glucose and insulin pathways promoted by an affluent adult environment which makes the offspring more susceptible to adult disease [25]. 

To study this phenomenon, several IUGR animal models have been developed, most commonly carried out in the monkey, pig, sheep, and rodents [26]. The animal models use different intervention strategies to cause IUGR and some of the most widely used methods include nutritional models with decreased caloric or protein intakes; surgical or utero-placental blood flow alterations such as uterine artery ligations; glucocorticoid treatment; and increased maternal stressors such as high heat [26]. These animal models have shown offspring to be IUGR, but do not exhibit the same adult disease manifestations, which may depend on the particular IUGR model utilized [27]. While these models have given insight into disease progression correlated with LBW, there is still much to be understood about the molecular pathways that can lead to adult disease. Recent studies have indicated telomere length and telomerase activity to be affected in various tissues, and this may affect disease progression and accelerated cellular aging in post-natal life.

## 3. Telomere Biology and Cellular Senescence

 Cellular senescence can be triggered by “critically” short or uncapped telomere(s) [28]. Telomeres are comprised of tandem DNA repeats (TTAGGG)^*n*^  found at the ends of chromosomes to protect them from inappropriate DNA fusions, DNA breaks and to prevent DNA shortening into coding DNA [29]. They protect DNA ends by forming a protective cap with a single strand telomere overhang and telomere binding proteins including TRF1, TRF2, and shelterin among others [30]. Telomeres are maintained in cells by the holoenzyme, telomerase. It consists of an enzymatic protein component, telomerase reverse transcriptase (TERT) and an RNA template component, telomerase RNA component (TERC). TERC is widely expressed, but TERT expression is tightly regulated and is only found highly expressed in germ cells, stem cells, and ~90% of cancer cell lines contain a functional telomerase [31]. Without a functional telomerase, a cell undergoing cell division will have progressive telomere shortening, resulting in telomere-dependent replicative senescence and an inability to divide further when a “critically” short telomere length is reached [32, 33]. Premature senescence can occur without critically short telomeres when cells encounter stressors including oxidative stress that can cause telomere dysfunction, telomere uncapping or other DNA damage. These stressors can trigger DNA damage response or senescent pathways, including p53-p21 ARF pathways and p16^INK4a^-Rb pathways [34, 35]. Average telomere length of circulating leukocytes has been shown to be a good indicator of aging and age-related diseases [36], but only one critically short telomere can initiate a DNA damage response to become senescent or apoptotic [37].

Telomerase may also exhibit other extra-telomeric functions involved in gene expression, DNA damage response, apoptosis regulation and a possible role in mitochondria and oxidative stress protection [38–40]. The role of telomerase in the mitochondria is an emerging topic, as recent studies have shown telomerase to translocate out of the nucleus to the mitochondria under oxidative stress conditions, leaving the genome vulnerable to telomeric DNA shortening and/or damage that could lead to premature cellular senescence [40]. Due to its role in apoptosis and senescence, telomerase/telomere biology has been explored for its roles in reproduction, placental development, and premature aging following IUGR [41–47]. These topics will be explored further in this paper. 

## 4. Functions of Telomeres and Telomerase in Germ Cells

 Germ cells are important as they determine the telomere length set for all the cells in our bodies and for the next generation [48]. Ovarian development is also greatly influenced by maternal nutrition and early growth, and it has been shown that undernutrition can affect ovarian size, follicular development and onset of puberty [49–52]. In the oocyte, high telomerase activity is present in the developing oocyte but significantly diminishes upon maturation [53, 54]. Once fertilization takes place telomerase activity dramatically rises, but is downregulated when cells go through differentiation [53–55]. To demonstrate the importance of telomerase activity in the oocyte, mice that are mTert^−/−^  have been used to show that it is imperative to have a functional telomerase enzyme for proper germ cell formation and fertility. Tert^−/−^ mice display infertility due to numerous causes that are present in the human populations including impaired meiotic synapsis and recombination, with more germ cells preferentially arresting in early meiosis [56, 57]. Similar problems can occur in human infertility, which may show a link between telomerase activity, telomeres, and germ cell health [58]. In fact, there appears to be a direct link between high telomerase activity in luteinised granulosa cells, higher rates of embryo implantation, and pregnancy during *in vitro* fertilization [42]. 

Today, women are delaying becoming pregnant and may also therefore encounter difficulties trying to conceive [59]. This decrease in fertility could be due, in part, to telomere dysfunction and DNA damage, as the germ cells have been exposed to a lifelong accumulation of possible reactive oxygen species (ROS)- induced damage upon the telomeric ends. Indeed, ROS-induced telomere shortening/uncapping has been observed in oocytes and/or preimplantation embryos leading to apoptosis or senescence at relatively long telomere lengths [60, 61]. Furthermore, oogenesis begins during early fetal life, where it can be exposed to an aged maternal environment. A recent paper has shown that maternal undernutrition can significantly affect primary, secondary, and antral follicle numbers in the adult rat offspring, with decreased factors involved in folliculogenesis, ovarian steroidogenesis, and ovulation [52]. Furthermore, female rats that have been exposed to undernutrition during development have been shown to go through reproductive senescence at an earlier age compared to the control counterparts [62]. It has been suggested these effects could be due to increased oxidative stress, as there was increased ovarian protein carbonyl content and hyperoxidized Prx3 [52]. Treatments with an antioxidant N-acetyl-L-cysteine (NAC) has been shown to delay oocyte aging, and increase oocyte quality and litter sizes [63]. Additionally, telomere length was elongated and telomerase activity greater in ovaries with this short term NAC treatment [63]. 

Stem cells are also susceptible to aging and ROS damage. In human and mice, telomeres shorten in stem cell populations as an organism ages [63, 64]. Over a lifespan, telomere shortening and increased oxidative stress can significantly reduce the self-renewal and proliferative capacity of a stem cell compartment [65, 66]. This has recently been shown to occur early in development, as stem cell populations from LBW infants show altered abilities compared to normal stem cells. Low birth weight endothelial colony forming progenitor cells (LBW-ECFCs) taken from cord blood have been shown to form fewer colonies compared to controls and take longer to appear, and show alterations in their ability to proliferate, migrate, and form sprouts and tubes *in vitro* [67]. LBW-ECFCs also displayed reduced capillary networks *in vivo* when injected in matrigel plugs in mice and was likely the result of an increase in gene expression of antiangiogenic genes including thrombospondin 1 (THBS1) compared to controls [67]. In addition, studies have found that there is a relationship between birth weight and the concentrations of endothelial progenitor cells in the cord blood, where LBW infants have the lowest concentrations compared to higher birth weight infants [68].

These studies suggest that telomere health may begin during germ cell development, when a fetus is still developing. These tantalizing outcomes show that an adverse intrauterine environment can negatively affect telomere health for future generations. 

## 5. Telomeres and Telomerase in the Placenta

 The placenta is an organ that is designed to be invasive into the maternal tissue to allow for an adequate supply of maternal blood for delivery of oxygen and nutrients to the fetus. To achieve this, extravillous trophoblast cells migrate and invade the maternal spiral artery walls in decidua and myometrium [69]. In the first weeks of pregnancy, there is rapid growth of the cells in a relatively hypoxic environment. Telomerase activity is the highest in placental trophoblast in the first trimester, and activity decreases through the duration of pregnancy to term [41, 70, 71]. Normal telomerase activity is correlated with HIF-1*α* expression, and it was shown to induce hTERT expression by binding to two HIF-1*α* consensus-binding sites in the hTERT promoter [72]. As the maternal arterial circulation is initiated, there is three-fold rise in intraplacental oxygen concentration [73], which could explain the normal decrease in telomerase activity through HIF-1*α* from 93.5% positive telomerase activity in first trimester chorionic villi versus 62.5% from second and third trimester chorionic villi [71]. Interestingly, in pregnancies complicated with IUGR and/or preeclampsia there is decreased telomerase activity and hTERT staining in placental trophoblasts compared to controls between 26 and 39 weeks [71, 74]. Additionally, decreased or absent telomerase activity can also be found in association with unfavourable outcomes of pregnancy such as spontaneous abortions and intrauterine fetal death [41]. Conversely, in hydatidiform molar pregnancies there is an increase in telomerase activity and hTERT staining that accompanies uncontrolled cell growth and division in the formation of a mass [70, 75].

To further demonstrate a relationship between placental telomerase activity and low birth weights, Kim and colleagues carried out a discordant twin study, where the smaller twin was found to have significantly decreased placental telomerase activity in relation to the normal birth weight twin [43]. Normal pregnancies were found to have long telomeres [76], and pregnancies complicated with IUGR and/or preeclampsia were found to have shortened placental telomeres compared to controls, but this shortening was not apparent in the cord blood [44, 74]. In association with telomere shortening, studies have found decreases in antiapoptotic protein Bcl-2 and upregulation of senescent markers p16 and p21 in the IUGR placenta [44]. These effects may be due to increases in oxidative stress markers in the cord blood and DNA damage in the IUGR placenta [77]. 

## 6. Telomeres and Telomerase in the**** Developmental Origins of Health**** and Disease

 Placental insufficiency resulting in LBW and IUGR can have a wide range of detrimental effects on a developing fetus, in addition to just being born small. Many studies are currently focusing on the “programming” or epigenetic modifications that can occur in utero and persist long term [78] but other disturbances are also taking place including increased oxidative stress which can have widespread effects. Interestingly, recent studies have implicated ROS in altering chromatin remodelling enzymes and histone modifications that control gene expression [79]. In humans, pregnancy complications including IUGR and preeclampsia have been shown to increase markers of oxidative stress and antioxidant defences in the mother and in neonates [80–83]. Oxidative stress can lead to lipid peroxidation, protein damage and DNA damage and can cause apoptosis if severe, or cells can exit the cell cycle and enter into premature senescence. There has been increasing evidence that cellular senescence contributes to the onset of disease, and is perhaps beginning *in utero.* Low birth weight studies in animals have indicated an increase in cell cycle inhibitors p16 and p21 along with alterations in the methylation and expression of p53 in various tissues including the kidney and pancreatic *β*-cells [45, 47, 84, 85]. Higher levels of ROS have been measured in these tissues, including an increase in the stress adaptor protein p66^Shc^, which has been shown to have significant impact on decreasing longevity [45, 86]. ROS in these tissues could contribute to DNA damage, and a study has shown a greater amount of DNA single strand breaks, shortened telomeres and increased oxidative stress in aortic tissues of maternal low protein recuperated pups [46]. This type of DNA damage can activate DNA damage responses, including the p16-Rb pathway and p53-p21 pathway to remove damaged cells from the replicating population to deter mutations from arising and being replicated. Telomere shortening or breaks can also turn on these responses in a cell, and studies have found an association between adverse intrauterine environment and shorter telomere length later in life in both human and animal studies [46, 87, 88]. Telomere attrition is also associated with cardiovascular disease and atherosclerosis [89–93] and type 2 diabetes [94–96].

**Figure 1:**
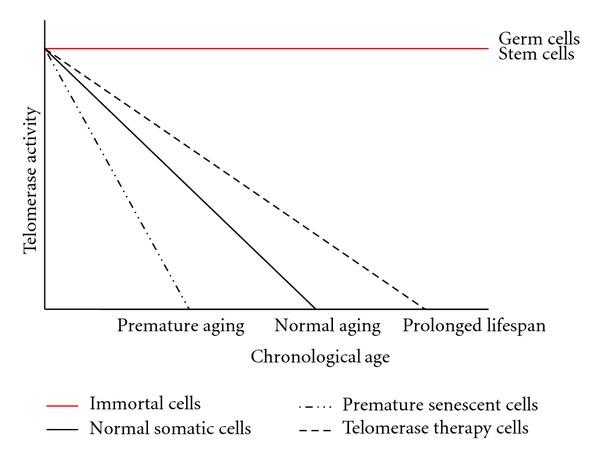
Telomere length and stability decrease throughout a lifespan. *In utero* stress can cause premature telomere shortening and destabilization that will cause cells to age and become senescent compared to normal individuals. This can be further exacerbated with poor post-natal nutrition. With new molecular modifiers of telomerase becoming available, it may soon be possible to rescue cells and reduce premature aging in a tissue. Cells that have a functional telomerase can maintain telomere integrity and be immortalized, such as stem cells and germ cells.

As a person ages, they acquire more senescent cells and accompanied by various aging pathologies including atherosclerosis [89]. If this accumulation begins at a younger age these senescent cells could not only intrinsically age organs more quickly, but they may affect neighbouring cells by secreting altered cytokines, extracellular matrix proteins, and growth factors that lead to further changes within aging organs and tissues [97]. Baker and colleagues designed an inducible transgenic system in a progeroid mouse model that eliminated p16 expressing senescent cells *in vivo*. The loss of senescent cells was sufficient to delay the onset of age-related pathologies in adipose, skeletal muscle, and eye tissue of these mice [98]. Furthermore, when this clearance of p16 expressing senescent cells occurred later in life, the progression of various aging diseases was attenuated [98]. This study demonstrates that the accumulation of senescent cells in tissues is involved in generating age-related organ pathologies and that their removal can prevent or delay tissue dysfunction and extend healthspan. This could be achieved with the maintenance of telomeres and telomerase activity to aid and repair oxidative stress and DNA damage, which could decrease senescent cells in a tissue (Figure 1). 

## 7. Perspectives

**Figure 2:**
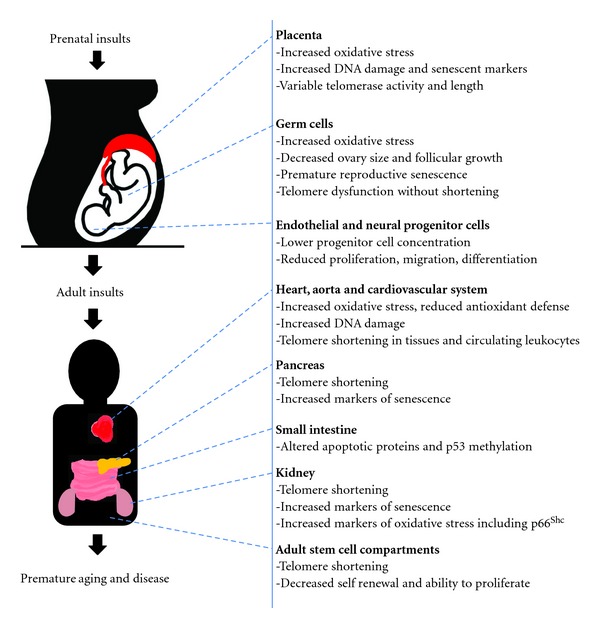
Various tissues in the body are affected by IUGR and have shown measurable changes in oxidative stress, telomerase activity, telomere shortening, and increased markers of senescence. These tissues can have a role in the development of adult diseases such as type 2 diabetes, cardiovascular disease, and metabolic syndrome.

 There is accumulating evidence that points to premature cellular aging as being a contributor to the poor postnatal outcomes of IUGR patients that can lead to increased risk for adult disease. Questions may arise whether early senescence onset is a cause or effect of IUGR? Nevertheless, telomere length and integrity is becoming a strong molecular marker of cellular aging in many studies including cardiovascular disease, type 2 diabetes, and overall health and longevity [95, 96, 99] (Figure 2). The shortened telomere lengths observed in young adults after intrauterine stress exposure [88] may predispose these individuals to early onset of various age-related diseases. Increased shortening of telomeres may be a result of the rapid postnatal catch up growth in tissues that have downregulated telomerase activity levels or due to the redistribution telomerase out of the nucleus in stressed tissues. Altered cellular metabolism is a hallmark of IUGR [100] and these metabolic shifts that elevate ROS production can increase telomere shortening or induce telomere dysfunction leading to premature senescence, [101] that may remain permanently damaged in a cell [102]. Conversely, telomere dysfunction itself induces altered metabolic and mitochondrial functions that may in turn cause further metabolic and oxidative stress deregulations in various tissues [103]. The induced metabolic syndrome observed in various IUGR models may be due to the early and increased accumulation of senescent cells within certain tissues. The pro/antioxidant balance is significantly affected in IUGR pregnancies [81, 83]. Postnatal antioxidant treatment with resveratrol prevents diet-induced metabolic syndrome in IUGR rats [104]. Future studies characterizing the role of the uterine environment on telomere and telomerase dynamics with premature senescence will lead to new knowledge on the etiology behind the developmental origins of health and disease leading to novel treatments such as telomerase therapy as a possible means to ameliorate the premature onset of adult disease in IUGR outcomes.
